# A Robust Method for Detecting Parking Areas in Both Indoor and Outdoor Environments

**DOI:** 10.3390/s18061903

**Published:** 2018-06-11

**Authors:** Wenhao Zong, Qijun Chen

**Affiliations:** Department of Control Science & Engineering, Tongji University, 4800 Cao’an Hwy, Shanghai 201804, China; 1310480@tongji.edu.cn

**Keywords:** image mosaic, computer vision, parking area detection

## Abstract

Although an automatic parking system has been installed in many vehicles recently, it is still hard for the system to confirm by itself whether a vacant parking area truly exists or not. In this paper, we introduced a robust vision-based vacancy parking area detecting method for both indoor and outdoor environments. The main contribution of this paper is given as follows. First, an automatic image stitching method is proposed. Secondly, the problem of environment illuminating change and line color difference is considered and solved. Thirdly, the proposed algorithm is insensitive to the shadow and scene diversity, which means the detecting result satisfies most of the environment. Finally, a vehicle model is considered for tracking and reconfirming the detecting results to eliminate most of the false positives.

## 1. Introduction

Since autonomous driving has become more and more popular in recent years, an automatic parking system, as one of autonomous driving’s most typical components, has become a hot topic both in research and volume manufacturing. Most of the automatic parking systems nowadays can only be called semi-automatic because a parking area detection method using ultra-sonic signals cannot even distinguish between a vacant parking area and a free space between two garbage bins. As a result, consumers need to activate the system close to the parking area and confirm the vacancy manually, which limits the application of this function. In this paper, a vision-based parking area detection method with the fusion of ultra-sonic sensors is proposed to solve the detecting problem. The reason why we choose vision is that a standard parking area always has specific marks around it, which makes up for the uncertainty of the ultrasonic method. Usually, the image is obtained from the fisheye cameras placed outside the vehicle, called a 360 surrounding view system [1]. In recent years, many works have been done using this typical system, such as image enhancing [2], lane marking detection [3], obstacle detection [4], etc.

## 2. Related Work

Some similar work has been done focusing on the parking area detecting and tracking problem. The authors of [5] proposed a surround view camera synthesis algorithm, but most attention has been paid to the color correction of the system only to makes the four images smoother for humans to see. The authors of [6] implemented the system mentioned in [5] on an emended system board. In the same year, The authors of [7] also gave a surrounding view camera solution for embedded systems, mainly focusing on the geometric and photometric alignment. Similarly, although a Harris corner detection [8] and BRIEF descriptor [9] based algorithm for images mosaicking is mentioned, not much detail was given, including the most important part of residual optimization. The authors of [10], from Delphi Automotive, proposed an image stitching method based on traditional checkerboard calibration and look-up tables (LUT). The obvious problem with this method is that a big check board is needed and the position of placement is strictly restricted. In addition, the number of cameras is not easy to expand for a bigger vehicle. The above works all focus on the image stitching problem without parking area detection.

**Figure 1:**
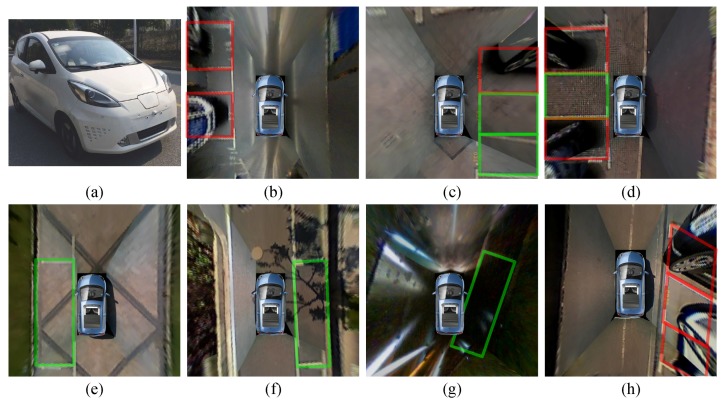
Typical scenes for vision-based parking area detection using green and red to represent vacancy or not. (**a**) Testing vehicle (**b**) Vertical parking areas in underground garage (**c**) Vertical parking areas on marble ground (**d**) Vertical parking areas with brick texture and similar line and ground color (**e**) Parallel parking areas on parquet ground (**f**) Parallel parking areas with strong shadow (**g**) Parallel parking areas at night (**h**) Oblique parking areas.

The authors of [11] proposed a visual-based free parking space detecting method. However, they only focused on the simplest situations with only white parallel lines on the ground and without mentioning the image stitching approach. The authors of [12] proposed a surrounding view-based parking area detection and tracking algorithm, but the method only works when the ground is clean without too many sundries or too much reflection of light like underground garages. In addition, the algorithm does not distinguish a vacancy, which will cause problems in practice. The authors of [13] introduced a complete system of using both a surround view system and an ultra-sonic method to obtain parking spaces and their availability. They paid more attention to the detection problem, but neglected the image stitching part. In addition, the paper only focused on an indoor environment without demonstrating any results on the special ground with much linear texture like brick stone ground. The authors of [14] introduced a method to detect available parking slots using around view monitor (AVM), which is similar to our 360 surrounding view system. However, they only tested the method in the place without ground reflection and without introducing their image stitching method. The authors of [15] introduced a guideline based detecting method, but they did not solve the overlap problem. In addition, they only tested their method in one environment. In conclusion, the difficulty of this problem is as follows, and is illustrated in Figure 1.

(i) The method to calibration surrounding cameras in order to form the bird view of the environment around the vehicle;
(ii) Due to the severe change of the color and luminance caused by reflection of the ground in garages, it is very hard to segment the image using RGB color.
(iii) Due to the great difference between the indoor and outdoor parking lots environment, it is very hard to train a learning based classifier or match with template. For example, the line color of the parking area can be any bright color compared with the ground color; the ground material and texture may different greatly from each parking lot; the shadow on the ground really does harm to the training accuracy, etc.

In this paper, the above difficulties are what mostly concern us. Solving these problems makes up of the biggest novelties of this paper. In Section 2, the system overview is demonstrated including sensor placement and a system diagram to give the reader a general idea of the system. In Section 3, a method for camera automatic calibration and image mosaic is proposed to obtain the image for parking area detection in the next few sections. In Section 4, a detailed algorithm for parking area detection and tracking in a single frame is introduced. In Section 5, a vehicle model-based parking area tracking and confirming method fusion with ultra-sonic are introduced. In Section 6 and Section 7, experiment results and the conclusion are demonstrated by tables and figures.

## 3. System Overview

**Figure 2:**
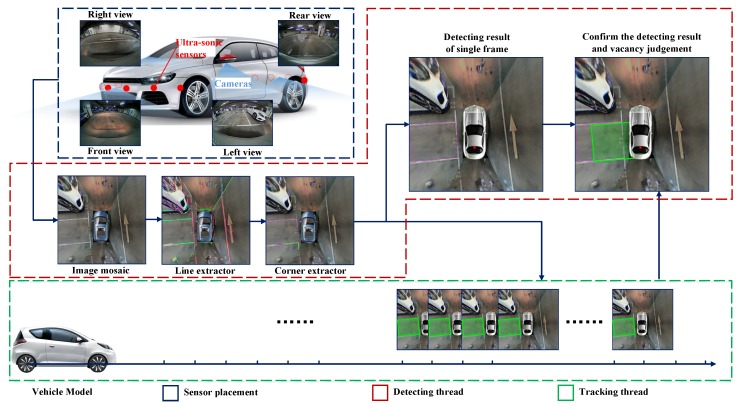
System diagram of parking area detection. Sensor placement is demonstrated in the dash box where the red dots stand for ultra-sonic and blue areas stand for the undistorted FOV of the surrounding cameras.

In this section, the sensor placement and a general procedure of the system will be introduced and demonstrated in Figure 2. The vehicle is equipped with four fisheye cameras with resolution of 640 × 480, horizontal and vertical field of view (FOV) of $194^{{}^{\circ}}$ and $134^{{}^{\circ}}$, respectively. The four cameras are placed in the front, at the rear and at two sides of the vehicle. Thanks to their large FOV, the overall view of the surrounding environment can be obtained without a blind point. However, the serious distortion of the view results in the great loss of the structured information such as the straight line, parallel and perpendicular. In addition, the vehicle is equipped with 12 ultra-sonic sensors with a maximum valid detection distance of 3.5 m. The FOV of the front and rear eight sensors are $100^{{}^{\circ}}$, and the side four sensors $60^{{}^{\circ}}$. Since the ultra-sonic is a simple and cheap sensor, it can only obtain the relative distance of the obstacle within the FOV without any angle information. As a result, it is used for fusion with the vision system to determine vacant areas. After four distorted images are obtained from the cameras, an undistortion procedure needs to be carried out. We use Apriltag [16], which is a kind of QR code, to calculate the homography matrix in order to obtain the bird’s eye view. Simultaneously, with two cameras seeing at least one identical Apriltag, the external parameters of cameras can be obtained automatically. These are all done automatically offline in the calibration stage. With the real-time panorama image calculated by the gained homography matrix, several steps will be operated to get the available parking area. There are two separate threads in this system: the detecting thread and tracking thread. In the detecting thread, the modified line extractor based on Line Segment Detector (LSD) [17] obtains the candidate parking slot line edges in the beginning. Secondly, the parking corner extractor obtained the structured information of the L-shaped components, which meets the specification of most of the parking areas. Thirdly, with the L-shaped result detected in the current frame and tracked from previous frames, a search method is proposed to obtained the candidate parking areas. In the tracking thread, a vehicle and Kalman Filter-based algorithm is used to update the real position of every parking area and give them a confidence score. Finally, with the help of an ultra-sonic and reconfirmation scheme, most of the false positives that include unparkable areas will be removed to obtain the final detecting result.

## 4. Surrounding Camera Image Stitching

In this section, a method for surrounding camera automatic panorama is proposed based on Apriltag—a practical and robust method of camera pose estimation. Since the surrounding cameras are fisheye ones with serious distortion, although wide vision is available, it is necessary to undistort the image before applying Apriltag for homography matrix calculation. Although there are many algorithms like [10,18,19,20,21] to get panorama images of a 360 surrounding view system, almost all of them need complex steps and big calibration fields. In addition, one of the biggest problems is that they are only suitable for four-camera calibration. If there is a truck or bus that needs more than four cameras, the method becomes invalid. So, a description of how to build a multi-camera automatic stitching system is given as follows. Assume the ground is planar and we have *n* cameras to calibrate. First, the intrinsic parameters of each camera are calibrated separately for image undistortion. Next, *n* big Apriltags are put approximately in the center of camera view on the ground and *n* small ones are put in the junction area that can be seen by two cameras. Finally, the panorama image will be output directly.

### 4.1. Apriltag

Apriltag [16] is an opensource robust and lightweight visual fiducial system. It is designed to recognize artificial landmarks. Each landmark has a full six degree of freedom (DOF) pose. This system includes four subsystems, a robust line detection system, a precious quadrangle detection system, a strong digital coding system, and a 6-DOF localization system. Many further applications can be developed based on this system. For example, its coding system can be used to generate user interfaces that overlay robots’ plans and task assignments onto a head-mounted display [22]. Its localization system can be used for a visual fiducial of the Visual-Inertial Motion Capture system [23], used to generate ground-truth robot trajectories and close control loops [24], or used to evaluate Simultaneous Localization and Mapping (SLAM) algorithms under controlled algorithms. Compared with previous methods (including ARTag [25] and Studierstube Tracker [26]), this visual fiducial has a small payload, but also can be detected and localized even though the original image has a very low resolution. The environment is at non-uniform illumination, the tag is oddly rotated and a large area is occluded and tucked away in a corner. In brief, it has a strong robustness to false positives arising from natural imagery and significantly higher localization accuracy than previous systems.

### 4.2. Image Stitching

**Figure 3:**
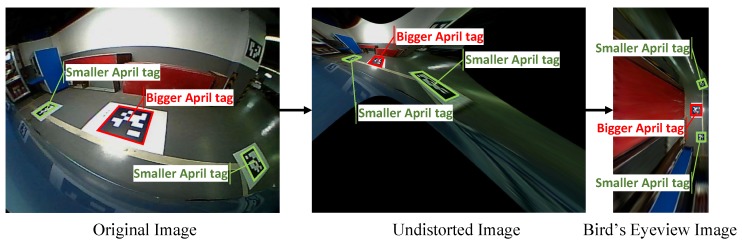
From left to right is the procedure of image undistortion and perspective transformation. The bigger Apriltags indicated with the red box are used to calculate the homography matrix. The smaller Apriltags indicated with the green box are used to montage images.

**Figure 4:**
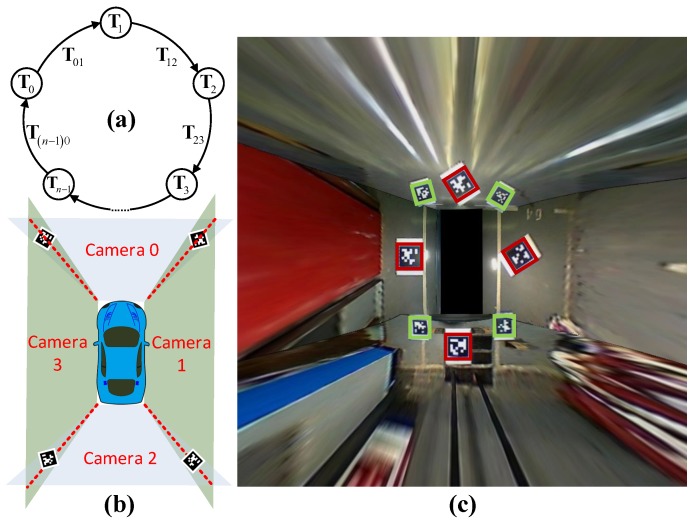
(**a**) General camera model. Nodes represent the positions of the cameras. Edges represent transferal of position from every two adjacent cameras. (**b**) Example of a four-camera panorama system. (**c**) Example panorama result of a four-camera system.

After *n* undistorted images are obtained, three Apriltags can be detected in one image, as is shown in Figure 3. The biggest one in the middle is what we choose to calculate homography. In this paper, the center of the rear axle of the vehicle is chosen to be the origin point $O_{W}$. The only thing needed for the measurement is the position of one of the *n* bigger Apriltags related to $O_{W}$. For the *i*th camera, four corner points of one Apriltag is enough to solve the Homography matrix $H_{i}$, which can transform the image to a bird’s eye view $I_{i}$ by equation $x^{\prime }=H_{i}x$, where x and $x^{\prime }$ are the pixel points before and after perspective transformation. We denote each corner point of *n* small Apriltags which can be seen by two cameras as $\left(u_{ij},v_{ij}\right)$, where *i* is the *i*th tag and j=0,1,2,3 means the index of the corner points. For the same point in two cameras, the distance error is defined by Equation (1).

(1) $${\mathrm{err}}_{i0}={∥{\left(u_{i0},v_{i0},1\right)}^{T}-T_{i}{\left(u_{\left(i+1\right)0},v_{\left(i+1\right)0},1\right)}^{T}∥}_{2}$$

where $T_{i}=\left(\begin{array}{ccc} \cos\Delta \theta_{i} & -\sin\Delta \theta_{i} & \Delta x_{i}\\ \sin\Delta \theta_{i} & \cos\Delta \theta_{i} & \Delta y_{i}\\ 0 & 0 & 1 \end{array}\right).\;\;\Delta x_{i},\Delta y_{i},$$\Delta \theta_{i}$ represent the translation and rotation values of camera *i* to camera i+1. Thus, mosaicking *n* cameras can be defined as an optimization problem with Equation (2), which actually minimizes the sum of all the distance errors.

(2) $$\underset{\Delta x_{i},\Delta y_{i},\Delta \theta_{i}}{\arg\min}\frac{1}{2}{∥p_{12n\times 1}-A_{12n\times 12n}{p^{\prime }}_{12n\times 1}∥}_{2}$$

where $p={\left(u_{00},v_{00},1,u_{01},v_{01},1,\ldots ,u_{\left(n-1\right)3},v_{\left(n-1\right)3},1\right)}^{T}$ and $p^{\prime }={\left(u_{10},v_{10},1,u_{11},v_{11},1,\ldots ,u_{03},v_{03},1\right)}^{T}$ are made up of all the corner points of smaller Apriltags. $A=diag\left(T_{0},\;T_{0},T_{0},T_{0},\ldots ,T_{n-1},T_{n-1},T_{n-1},T_{n-1}\right)$ is a diagonal matrix consists of the 3×3 rotation and translation matrices of cameras adjacent to each other. After Equation (2) is solved, the prior that *n* cameras form a closed loop is used to average the residual to every node. Here, the left-top point of $I_{0}$ and its orientation according to the vehicle $O_{W}$ is denoted by $\xi_{0}=\left(x_{0W},y_{0W},\theta_{0W}\right)$. For each $\xi_{i}$ and $\xi_{i+1}$, the error matrix can be defined by Equation (3), and the optimization function is defined by Equation (4). The overall stitching procedure is demonstrated in Figure 4.

(3) $$\begin{array}{cc} e_{i} & =B\cdot b\\ B & =\left(\begin{array}{cccc} \cos\Delta \theta_{i} & -\sin\Delta \theta_{i} & 0 & -\Delta x_{i}\\ \sin\Delta \theta_{i} & \cos\Delta \theta_{i} & 0 & -\Delta y_{i}\\ 0 & 0 & 1 & 0\\ 0 & 0 & 0 & 1 \end{array}\right)\\ b & ={\left(x_{\left(i+1\right)W}-x_{iW},y_{\left(i+1\right)W}-y_{iW},\theta_{\left(i+1\right)W}-\theta_{iW},1\right)}^{T} \end{array}$$

(4) $$\underset{x_{iW},y_{iW},\theta_{iW}}{\arg\min}\frac{1}{2}{e_{i}}^{T}e_{i}$$

## 5. Parking Space Detection in a Single Frame

In this section, parking area detection in a single frame contains the following subsections, including a modified line extractor based on LSD, L-shaped corner extractor for parking area entry point detection and a parking area searching method. The reason why we choose a line extractor rather than a traditional color segmentation method is that the gradient-based method is more robust for a specific color threshold, since the line color of the parking area in a single frame may differ greatly, causing a luminance change or shadow. An L-shaped structure is the most common style of the parking area line mark in the world. By locating the L-shaped corners, parking areas can be searched though proper combinations.

### 5.1. Line Extractor

**Figure 5:**
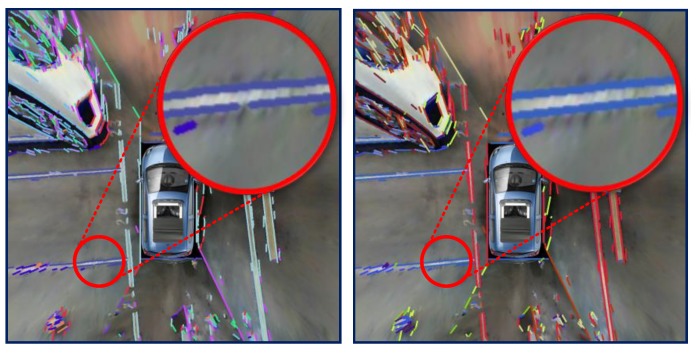
Result of line combination **Left**: before combination **Right**: after combination.

**Figure 6:**
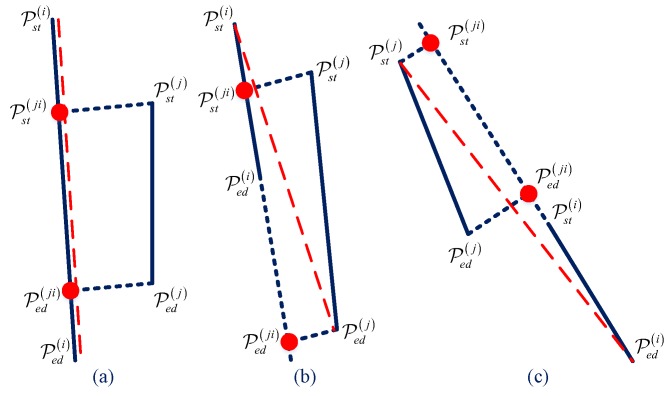
Three positions of two lines with red dashed lines indicating the line combination result (**a**) containing (**b**) overlap (**c**) disjoint.

**Figure 7:**
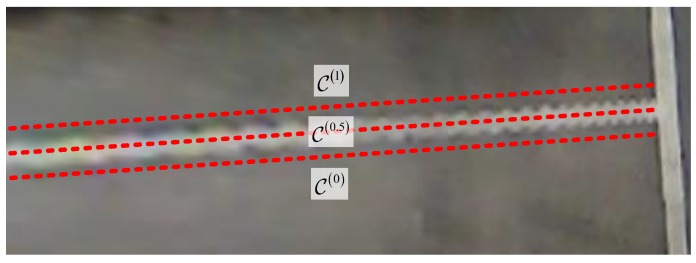
Method to determine the candidate line edge by average pixel gray value.

One familiar line extractor method is known as Hough Transform (HT) [27], but HT performs well only when the line is straight and long. The situation in this paper is different as the parking area line marks may be short and discontinuous. Due to the poor quality of the on-vehicle camera and the changeable environment, HT easily fails, and is quite hard to adjust parameters. Therefore, a gradient based line descriptor is chosen to solve the problem. For the mosaic image $I^{\left(t\right)}$ obtained at time *t*, a procedure including converting RGB color to gray scale, erode and dilate with a median filter is applied to the source image to get the preprocessed image denoted as $I_{pre}^{\left(t\right)}$. Afterwards, an LSD descriptor is applied to $I_{pre}^{\left(t\right)}$ with default parameters to obtained a set of line segment L. For each line $L^{\left(i\right)}$ in L, $P_{st}^{\left(i\right)}\left\{x,y\right\}$ and $P_{ed}^{\left(i\right)}\left\{x,y\right\}$ stands for the start and end point of $L^{\left(i\right)}$. Here, the start point is set to the left of or above the end point by $\mathrm{swap}\left(P_{st}^{\left(i\right)},P_{ed}^{\left(i\right)}\right)$ when $P_{st}^{\left(i\right)}.x>P_{ed}^{\left(i\right)}.x$ or $P_{st}^{\left(i\right)}.x=P_{ed}^{\left(i\right)}.x$ and $P_{st}^{\left(i\right)}.y>P_{ed}^{\left(i\right)}.y$. The angle of the $i^{th}$ line segment is denoted by $\alpha^{\left(i\right)}$. The L is divided into 20 groups by an angle which is denoted by $\left\{L_{0},L_{1},\cdots ,L_{19}\right\}$. For every two lines $L_{m}^{\left(i\right)}$ and $L_{m}^{\left(j\right)}$ in groups $L_{m}$, the $\mathrm{Dis}\left(L_{m}^{\left(i\right)},L_{m}^{\left(j\right)}\right)$ is calculated to estimate if $L_{m}^{\left(i\right)}$ and $L_{m}^{\left(j\right)}$ need to be combined into a new line, which is defined by the following Equation (5) and demonstrated in Figure 5.

(5) $$\mathrm{Dis}\left(L_{m}^{\left(i\right)},L_{m}^{\left(j\right)}\right)=0.5\left(\mathrm{Dis}\left(P_{st}^{\left(i\right)},L^{\left(j\right)}\right)+\mathrm{Dis}\left(P_{st}^{\left(j\right)},L^{\left(i\right)}\right)\right),$$

where $\mathrm{Dis}\left(P_{st}^{\left(i\right)},L^{\left(j\right)}\right)$ is the distance from point $L_{st}^{\left(i\right)}$ to line $L^{\left(j\right)}$. Here, three positions of two lines are defined as: containing, overlap and disjoint, which is demonstrated in Figure 6. The position is used to determine whether two line segments $L_{m}^{\left(i\right)}$ and $L_{m}^{\left(j\right)}$ need to combine or not. In situation (a) and (b), if $\mathrm{Dis}\left(P_{st}^{\left(i\right)},L^{\left(j\right)}\right)<\omega$ which is a small threshold of the line distance, combination is needed. In situation (c), if $\mathrm{Dis}\left(P_{st}^{\left(i\right)},P_{ed}^{\left(ji\right)}\right)<\mu$ which is a small threshold of the point distance, combination is also needed. The combination result is to renew $L_{m}^{\left(i\right)}$ to a line segment between two farthest points chosen from $P_{st}^{\left(i\right)}$, $P_{ed}^{\left(i\right)}$, $P_{st}^{\left(j\right)}$, $P_{ed}^{\left(j\right)}$, $P_{st}^{\left(ji\right)}$ and $P_{ed}^{\left(ji\right)}$. After the procedure of combination is finished, the line width threshold $\eta_{min}$ and $\eta_{max}$ is used for deleting very far away line tuples, and the line groups are renewed to the line tuple which $\left\{L_{m}^{\left(i\right)},L_{m}^{\left(j\right)}\right\}$ may be the candidate edges of line marks. Finally, the color change from inside of the line tuple to outside will be taken into consideration. In this paper, we assume that the color of the parking area line mark is brighter than the ground color. Thus, the color of the middle line of the tuple $\left(L_{m}^{\left(0\right)},L_{m}^{\left(1\right)}\right)$ is compared with the one out of the tuple, which is defined in Equation (6) and demonstrated in Figure 7,

(6) $$\begin{array}{c} \left|\frac{1}{n}\sum_{i=0}^{n-1}C^{\left(0\right)}\left\{x_{i},y_{i}\right\}-\frac{1}{m}\sum_{i=0}^{m-1}C^{\left(1\right)}\left\{x_{i},y_{i}\right\}\right|<\varepsilon ,\\ \left|\frac{1}{n}\sum_{i=0}^{n-1}C^{\left(0\right)}\left\{x_{i},y_{i}\right\}-\frac{1}{k}\sum_{i=0}^{k-1}C^{\left(0.5\right)}\left\{x_{i},y_{i}\right\}\right|>\xi , \end{array}$$

where m,n,k∈Z, $C^{\left(0.5\right)}\left\{x_{i},y_{i}\right\}$ is the gray pixel value on middle line of the tuple at position $\left(x_{i},y_{i}\right)$ and $C^{\left(0/1\right)}\left\{x_{i},y_{i}\right\}$ is the gray pixel value on two sides of the candidate line edges. The distance between two adjacent lines equals $\mathrm{Dis}\left(L_{m}^{\left(0\right)},L_{m}^{\left(1\right)}.\right)$. ε and ξ are color thresholds, where ε is close to zero, and ξ needs to be as large as possible, theoretically.

### 5.2. L-Shaped Corner Extractor

**Figure 8:**
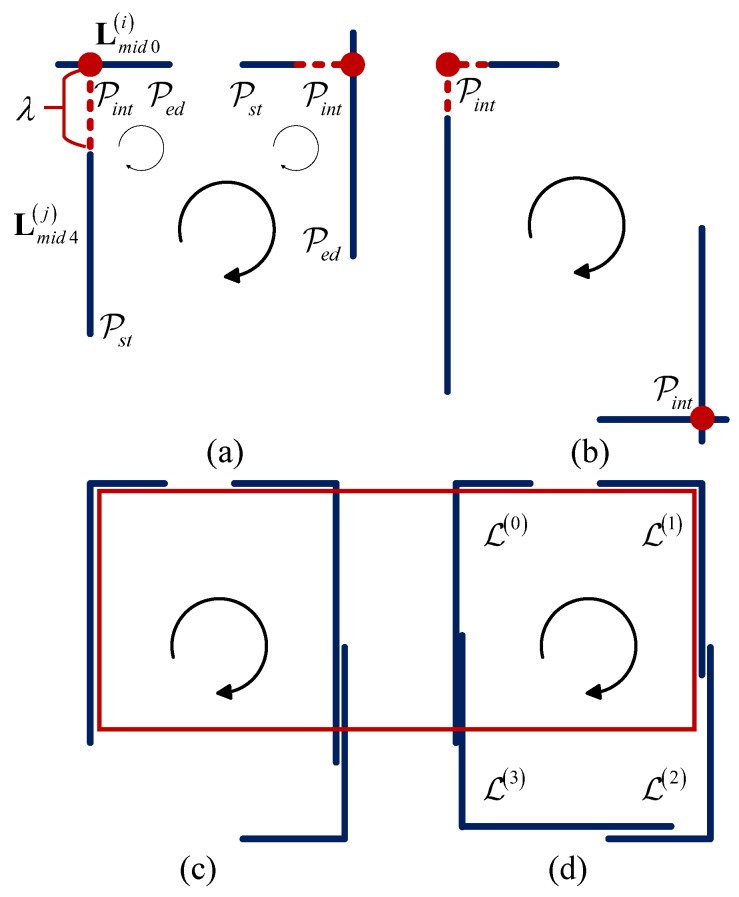
Four situations that can form an L-shaped structure are demonstrated in (**a**,**b**). The red dots represent the virtual or actual intersections of two lines with distance tolerance value λ. The attribution $P_{\mathrm{st}},P_{\mathrm{int}},P_{\mathrm{ed}}$ of $L^{\left(i\right)}$ are always clockwise. Four situations of L-shaped structure to form temp parking spaces are demonstrated in sub-figures (**a**–**d**). In sub-figure (**d**), L-shaped structures in each temp parking space are also clockwise. The red rectangle in (**c**,**d**) is the situation that an error detection occurred without removing an L-shaped structure between spaces.

From the above subsections, candidate parking area edged lines are detected and represented in the tuple set $\left\{\left(L_{0}^{\left(0\right)},L_{0}^{\left(1\right)}\right),\left(L_{1}^{\left(0\right)},L_{1}^{\left(1\right)}\right),\ldots ,\left(L_{m}^{\left(0\right)},L_{m}^{\left(1\right)}\right)\right\},m\in Z$, which means there are *m* probable parking area line marks in $I^{\left(t\right)}$. Considering that an L-shaped structure is the key feature of the parking area, the method to detect and locate it is what we focus on in this subsection. First, a T-shaped structure should be detected, which is the base of the L-shaped one. Each element of the tuple set is replaced by the middle line in order to form the new tuple with proper intersection angle. The original line set arranged by angle is now replaced by the middle line of the parking area line mark denoted as $\left\{L_{mid0},L_{mid1},\cdots ,L_{mid19}\right\}$. The segments are grouped by angle to accelerate the search for the segment with proper intersection angle. In addition, throughout the above operation, each element $L_{i}$ contains only the most probable line marks. In this paper, considering most of the situations in China, we choose the angle difference of two searching group to be $54^{{}^{\circ}}$, $90^{{}^{\circ}}$ and $126^{{}^{\circ}}$. Without losing generality, we use $L_{mid0}^{\left(i\right)}$ and $L_{mid4}^{\left(j\right)}$ to introduce the algorithm of an L-shaped corner extractor. Four situations are illustrated in Figure 8a,b distinguished from each other by the position of the intersection point $P_{int}^{\left(ij\right)}\left(x,y\right)$ of $L_{mid0}^{\left(i\right)}$ and $L_{mid4}^{\left(j\right)}$.

- In situation (a), $P_{int}^{\left(ij\right)}\in L_{mid4}^{\left(i\right)}$ and $P_{int}^{\left(ij\right)}\notin L_{mid0}^{\left(j\right)}$. If
  (7) $$\begin{array}{c} \max\left(\mathrm{Dis}\left(P_{int}^{\left(ij\right)},P_{st0}^{\left(i\right)}\right),\mathrm{Dis}\left(P_{int}^{\left(ij\right)},P_{ed0}^{\left(i\right)}\right)\right)>\tau ,\\ \min\left(\mathrm{Dis}\left(P_{int}^{\left(ij\right)},P_{st0}^{\left(i\right)}\right),\mathrm{Dis}\left(P_{int}^{\left(ij\right)},P_{ed0}^{\left(i\right)}\right)\right)<\lambda , \end{array}$$
  judging if $\mathrm{Dis}\left(P_{int}^{\left(ij\right)},P_{st4}^{\left(j\right)}\right)>\tau$, make an L-shaped tuple $L^{\left(k\right)}\left\{\left\{P_{int}^{\left(ij\right)},P_{st0}^{\left(i\right)}\right\},\left\{P_{int}^{\left(ij\right)},P_{ed4}^{\left(j\right)}\right\}\right\}$, where τ is the minimum length of one parking side. Usually, this value is smaller than the reality because not all of the four sides are closed. λ stands for the maximum gap tolerance from the intersection point to the nearest end point of $L_{mid0}^{\left(i\right)}$.
- Situation (b) is similar to (a).
- In situation (c), $P_{int}^{\left(ij\right)}\notin L_{mid4}^{\left(i\right)}$ and $P_{int}^{\left(ij\right)}\notin L_{mid0}^{\left(j\right)}$. If
  (8) $$\begin{array}{c} \max\left(\mathrm{Dis}\left(P_{int}^{\left(ij\right)},P_{st0}^{\left(i\right)}\right),\mathrm{Dis}\left(P_{int}^{\left(ij\right)},P_{ed0}^{\left(i\right)}\right)\right)>\tau ,\\ \max\left(\mathrm{Dis}\left(P_{int}^{\left(ij\right)},P_{st4}^{\left(i\right)}\right),\mathrm{Dis}\left(P_{int}^{\left(ij\right)},P_{ed4}^{\left(i\right)}\right)\right)>\tau ,\\ \min\left(\mathrm{Dis}\left(P_{int}^{\left(ij\right)},P_{st0}^{\left(i\right)}\right),\mathrm{Dis}\left(P_{int}^{\left(ij\right)},P_{ed0}^{\left(i\right)}\right)\right)<\lambda ,\\ \min\left(\mathrm{Dis}\left(P_{int}^{\left(ij\right)},P_{st4}^{\left(i\right)}\right),\mathrm{Dis}\left(P_{int}^{\left(ij\right)},P_{ed4}^{\left(i\right)}\right)\right)<\lambda , \end{array}$$
  add a new tuple $L^{\left(k\right)}\left\{\left\{P_{int}^{\left(ij\right)},P_{st0}^{\left(i\right)}\right\},\left\{P_{int}^{\left(ij\right)},P_{ed4}^{\left(j\right)}\right\}\right\}$ to L-shaped set.
- In situation (d), $P_{int}^{\left(ij\right)}\in L_{mid4}^{\left(i\right)}$ and $P_{int}^{\left(ij\right)}\in L_{mid0}^{\left(j\right)}$. The distance of $P_{int}^{\left(ij\right)}$ to each end point of $L_{mid0}^{\left(i\right)}$ and $L_{mid4}^{\left(j\right)}$ needs to be calculated. If $\mathrm{Dis}\left(P_{int}^{\left(ij\right)},P_{st0}^{\left(i\right)}\right)>\tau$ and $\mathrm{Dis}\left(P_{int}^{\left(ij\right)},P_{ed4}^{\left(j\right)}\right)>\tau$, add a new tuple $L^{\left(k\right)}\left\{\left\{P_{int}^{\left(ij\right)},P_{st0}^{\left(i\right)}\right\},\left\{P_{int}^{\left(ij\right)},P_{ed4}^{\left(j\right)}\right\}\right\}$ to L-shaped set.

### 5.3. Candidate Parking Area Searching Method

After the L-shaped set $L\left\{L^{\left(0\right)},L^{\left(1\right)},L^{\left(2\right)},\ldots ,L^{\left(m\right)}\right\}$, m∈Z is obtained, parking areas containing at least two L-shaped structures will be extracted. In this subsection, a searching algorithm is proposed for the candidate parking space detection. At first, a four-L-shaped structure is used to represent a temp parking space denoted by $tpksps^{\left(i\right)}\left\{L^{\left(0\right)},L^{\left(1\right)},L^{\left(2\right)},L^{\left(4\right)}\right\}$ , in which $L^{\left(0\right)}$ to $L^{\left(3\right)}$ are anti-clockwise, and then parallelogram constrain is applied to solve the final position of four parking points denoted by $pksps^{\left(i\right)}\left\{P^{\left(0\right)},P^{\left(1\right)},P^{\left(2\right)},P^{\left(4\right)}\right\}$.

As is demonstrated in Algorithm 1, for every two elements $L^{\left(i\right)},L^{\left(j\right)}\in L$, it is necessary to judge whether they can form a new temp parking space or should be added to the existing one in array tpksps. Thus, Function IsNewTempPkSp proposed with current L-shaped structure $L^{\left(i\right)}$, the number of temp parking spaces *n* as input and tpksps as both input and output. Two initial position relations of $L^{\left(i\right)}$ and $L^{\left(j\right)}$ are defined to be the possible parking spaces by Equations (9) and (10), where $\vec{st_{i}}$ is the vector from $L^{\left(i\right)}.p_{\mathrm{int}}$ to $L^{\left(i\right)}.p_{\mathrm{st}}$, ξ is a fault-tolerant value close to zero, $\mathrm{Angle}\left(\cdot \right)$ returns vector angle $\in \left[0,180\right]$, $\mathrm{Dis}\left(\cdot \right)$ returns point-point, point-line and line-line distance, according to its input value type. Both $L^{\left(i\right)}$ and $L^{\left(j\right)}$ are needed for comparison with all the detected temp parking spaces. The *i*th temp parking space $tpklots\left[i\right]$ may consist of two, three or four L-shaped structures. According to the pre-defined position relationship above, only if $L^{\left(i\right)}$ satisfies every L-shaped structure in $tpklots\left[i\right]$ can it be added to $tpklots\left[i\right]$, otherwise $L^{\left(i\right)}$ and $L^{\left(j\right)}$ forms a new temp parking space. In addition, to avoid the false detection in Figure 8c,d, it is necessary to make sure that there is no other $L^{\left(k\right)}$ between $L^{\left(i\right)}$ and $L^{\left(j\right)}$ almost on the same line, which is defined by Algorithm 2 IsNewTempPkSp. After finishing searching for all of the elements in L, real parking spaces with parallelogram constrain will be calculated by their center *c*, width *w*, length *l*, acute angle of parking space α, angle of positive *x*-axis and first side parallel with it through clockwise spinning θ. In Figure 8a, center point *c* is estimated by using the minimum parking length.

- situation $L^{\left(i\right)},L^{\left(j\right)}$ adjacent
  (9) $$\begin{array}{c} \mathrm{Angle}\left(\vec{st_{i}},\vec{ed_{j}}\right)-\pi =\xi \\ \mathrm{Angle}\left(\vec{ed_{i}},\vec{st_{j}}\right)=\xi \\ \mathrm{Dis}\left(\vec{st_{i}},\vec{ed_{j}}\right)=\xi \\ \mathrm{Dis}\left(L^{\left(i\right)}.p_{\mathrm{int}},L^{\left(j\right)}.p_{\mathrm{int}}\right)⩽\mathrm{Dis}\left(L^{\left(i\right)}.p_{st},L^{\left(j\right)}.p_{ed}\right) \end{array}$$
- situation $L^{\left(i\right)},L^{\left(j\right)}$ opposite
  (10) $$\begin{array}{c} \mathrm{Angle}\left(\vec{st_{i}},\vec{st_{j}}\right)-\pi =\xi \\ \mathrm{Angle}\left(\vec{ed_{i}},\vec{ed_{j}}\right)=\xi \\ \mathrm{Dis}\left(L^{\left(i\right)}.p_{\mathrm{int}},L^{\left(j\right)}.p_{\mathrm{int}}\right)⩾\mathrm{Dis}\left(L^{\left(i\right)}.p_{st},L^{\left(j\right)}.p_{st}\right)\\ \mathrm{Dis}\left(L^{\left(i\right)}.p_{\mathrm{int}},L^{\left(j\right)}.p_{\mathrm{int}}\right)⩾\mathrm{Dis}\left(L^{\left(i\right)}.p_{ed},L^{\left(j\right)}.p_{ed}\right) \end{array}$$

**Algorithm 1:** algorithm parking space search.

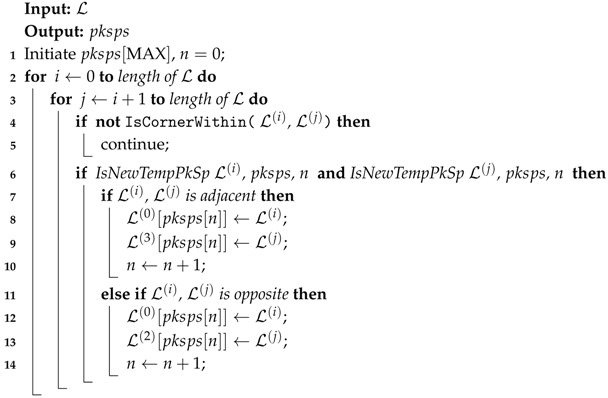



**Algorithm 2:** IsNewTempPkSp(*L*, pksps, *n*).

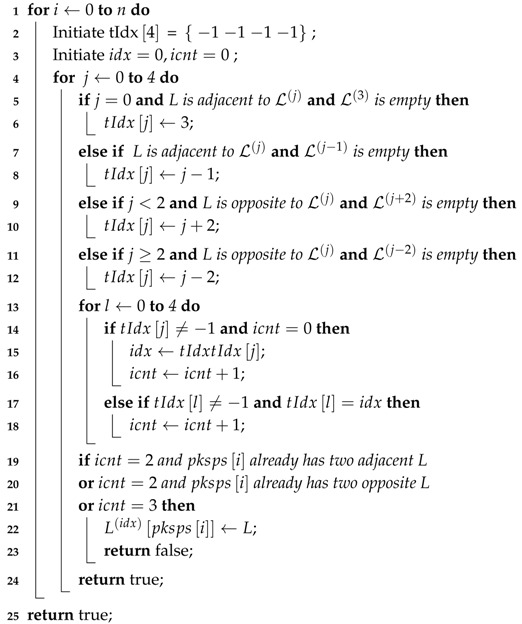



## 6. Parking Space Tracking and Parkable Confirmation

In this section, a vehicle kinematics model [28] and Kalman Filter [29] based parking space tracking algorithm is proposed, which can be divided into three parts: introduction of vehicle model, a method to observe parking space on the premise of single frame detection results obtained above and a confidence level calculating scheme. In addition, parkable confirmation method of every detecting result is introduced as the last part of this section.

### 6.1. Vehicle Model

In this subsection, we introduce a vehicle model, which measures the vehicle speed *v* and steering wheel angel δ with fixed intervals Δt, typically at 100 hertz. It assumes Gaussian noise *q* for vehicle speed and steering wheel angle measurement. This can be formulated by Equation (11),

(11) $$v=\tilde{v}+q_{v},\delta =\tilde{\delta }+q_{\delta }$$

**Figure 9:**
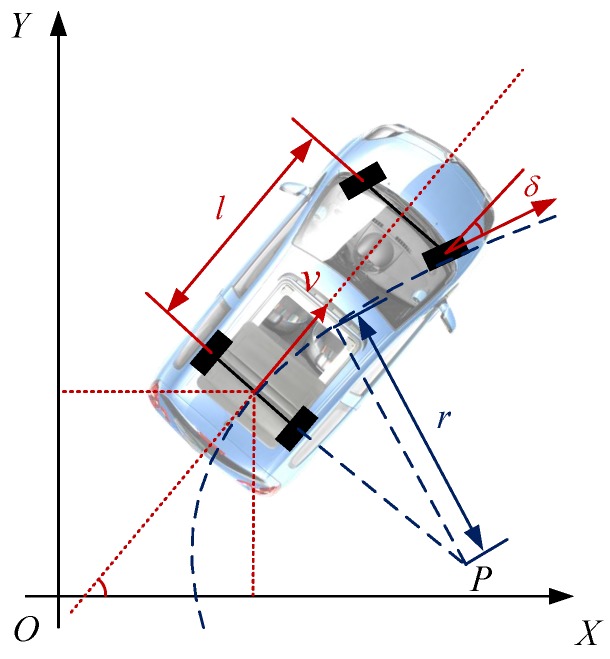
Ackermann vehicle model. *l* is the vehicle wheel base. *v* is velocity of rear axle center. δ is the tire angel. P is the instantaneous center of rotation.

Since vehicles always move a short distance when the system is trying to initialize, we assume the motion of the vehicle in initialization procedure is planar. Based on Ackermann steering geometry, the center *P* of circular is known as Instantaneous Center of Rotation. This can be formulated in Equation (12) and shown in Figure 9

(12) $$\left\{\begin{array}{c} \delta =\tau \psi \\ r=l/\tan\delta \\ \Delta \theta =v\Delta t/r\\ \Delta X=r\left(1-\cos\left(\Delta \theta \right)\right)\\ \Delta Y=r\sin\left(\Delta \theta \right) \end{array}\right.,$$

where ψ is the steering angle measurement. τ is transmission ratio from steering wheel to tire angle whose value is usually around 1/16. *r* is instantaneous radius of the rotation. *v* is the velocity of rear axle center. Δθ is the heading increment in a period of Δt. ΔX and ΔY are the lateral and longitudinal distance increment relative to Δθ.

### 6.2. Parking Space Tracker

The vehicle kinematics model is the basic model to describe vehicle motion. Considering the cumulative error of the vehicle model, it is unreliable to use only this information to estimate the real position of each parking space. Thus, measurement data is introduced to stabilize the system by Kalman Filter. Measurement data consists of two situations, namely, the single frame detection mentioned above and estimation from one L-shaped structure. Since the center, angle and size of each parking space has been obtained, it is possible to use only one L-shaped structure to estimate the updated position. This is useful, especially when the L-shape is obscured during a parking procedure. Therefore, there are in total five situations in which a parking space is detected corresponding to the number of L-shaped structure and their position relationship, respectively. With the vehicle kinematics model, a parking space can be updated by Equation (13)

(13) $$\begin{array}{c} c_{2\times \left(N+M\right)}=R_{2\times 2}\left(c_{2\times N}-T_{2\times N}\right)\cup {\hat{c}}_{2\times M},\\ \theta_{N+M}=\left(\theta_{N}-1_{N}\cdot \Delta \theta \right)\cup {\hat{\theta }}_{M}, \end{array}$$

where c is the 2×N center point matrix in former frames. $\hat{c}$ is the newly detected 2×M center point matrix in the current frame different from c. R is the 2×2 rotation matrix $\left[\begin{array}{cc} \cos\left(\Delta \theta \right) & -\sin\left(\Delta \theta \right)\\ \sin\left(\Delta \theta \right) & \cos\left(\Delta \theta \right) \end{array}\right]$. T is the 2×1 translation matrix ${\left[\Delta X,\Delta Y\right]}^{T}$, Δt is the processing period.

Observation is introduced when the same parking space is detected. In the section, the Extended Kalman Filter (EKF) is used to track the vehicle position. With the incrementation between two update periods, ΔX,ΔY,Δθ can be obtained to update the position of every parking space. The prediction model is given in Equations (14) and (15).

(14) $$\mu_{t}=\mu_{t-1}+F_{x}^{T}\left(\begin{array}{c} -\frac{l}{\tan\left(\tau \psi \right)}\left(\sin\left(\theta \right)+\sin\left(\theta +\frac{v\Delta t}{l}\tan\left(\tau \psi \right)\right)\right)\\ \frac{l}{\tan\left(\tau \psi \right)}\left(\cos\left(\theta \right)-\cos\left(\theta +\frac{v\Delta t}{l}\tan\left(\tau \psi \right)\right)\right)\\ \frac{v\Delta t}{l}\tan\left(\tau \psi \right) \end{array}\right),$$

(15) $${\bar{\Sigma }}_{t}=G_{t}\Sigma_{t-1}G_{t}^{T}+F_{x}^{T}R_{t}^{x}F_{x},$$

where $\mu_{t}={\left[p_{0},p_{1},\ldots ,p_{n},x_{v}\right]}^{T}$ is state vector, $x_{v}=\left[x,y,\theta \right]$ is vehicle state, $p_{i}={\left[c_{x},c_{y},\varphi ,w,l\right]}^{T}$ is the $i^{th}$ parking space, $F_{x}=\left[I_{3\times 3},0_{3\times 5N}\right]$, $G_{t}=\left(\begin{array}{cc} \frac{\partial \left(\mu_{t}\right)}{\partial x_{v}^{T}} & 0\\ 0 & I_{5N\times 5N} \end{array}\right)$, ${\bar{\Sigma }}_{t}$ is predicted covariance. The correction procedure is given as follows. If the parking area *i* did not appear before, its position can be calculated by (13). If it is already in the map, the observation equation is defined by Equation (16)

(16) $${\hat{z}}_{t}^{i}=\left(\begin{array}{c} \begin{array}{c} \left(p_{i}\left[x\right]-x_{v}\left[x\right]\right)\cos\left(x_{v}\left[\theta \right]\right)-\left(p_{i}\left[y\right]-x_{v}\left[y\right]\right)\sin\left(x_{v}\left[\theta \right]\right)\\ \left(p_{i}\left[x\right]-x_{v}\left[x\right]\right)\sin\left(x_{v}\left[\theta \right]\right)+\left(p_{i}\left[y\right]-x_{v}\left[y\right]\right)\cos\left(x_{v}\left[\theta \right]\right) \end{array}\\ p_{i}\left[\theta \right]-x_{v}\left[\theta \right]\\ \begin{array}{c} p_{i}\left[w\right]\\ p_{i}\left[l\right] \end{array} \end{array}\right),$$

where ${\hat{z}}_{t}^{i}$ is the observation vector, $p_{i}$ and $x_{v}$ are the state vectors mentioned above. With the observation equation, Kalman gain $K_{t}^{i}$ can be calculated with the classic EKF model, by taking a partial derivative with respect to the state variables. Through traversal all of the detected parking areas, the predicted state ${\bar{\mu }}_{t}$ and covariance ${\bar{\Sigma }}_{t}^{i}$ are updated in each iteration. In practical application, the number of historical parking spaces *N* are limited to a small quantity to ensure a low computational cost.

### 6.3. Parkable Area Detection

**Figure 10:**
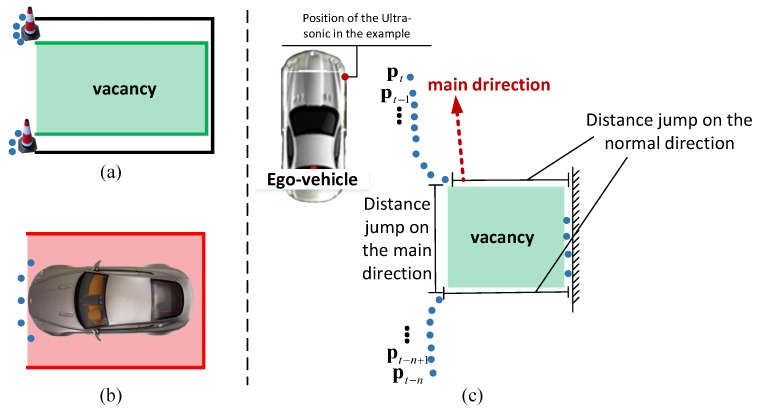
Parkable detection with an ultra-sonic sensor. In (**a**), the black box is the vision detecting result. By fusion with sonic data, the parkable area shrinks to the green area. In (**b**), the parking area detected by the camera has a car parking in it. Therefore, the result needs to be abandoned. In (**c**), vacancy area is obtained by taking the example of the right-front sensor. The blue points are the historical points obtained by the right-front sensor integrated over time.

If only parking lines are taken into consideration, it is impossible to decide whether the detecting result is vacant or not. If we choose a pure visual method to train a vacancy model, the algorithm will be very time consuming and need a powerful CPU or even GPU. In addition, the ground situation is very complicated, such as random light reflection, texture and color caused by different ground material and unknown objects rather than vehicles inside the parking area, etc. Even if we have a big number of training samples, it is easy to cause over-fitting since the samples have too much noise. As a result, sensor fusion is our best choice. We use the ultra-sonic method to judge if the visual detecting results are parkable according to the nearest obstacle distance to the side of the ego vehicle. Through integration vehicle speed over time, the discrete distance obtained by a single side sonic sensor can form the tendency of the obstacles next to the ego vehicle. The visual detecting results will be shrunk or abandoned with consideration of obstacle positions, which is demonstrated in Figure 10.

Here, the obstacle distance obtaining from the ultra-sonic is modeled as a point denoted by $p_{t}={\left[x,y\right]}^{T}$, where *t* is current time stamp, *x* is the distance to the obstacle, *y* is the longitudinal distance from the original point of the vehicle. Without losing the generality, we take the right front sonic sensor as an example. Assume we already have the set of historical points obtained from t−n to t−1 denoted by $P_{t-1}=\left[p_{t-n},p_{t-n+1},\ldots ,p_{t-1}\right]$. The update method of the obstacle distance obtain from the sensor is denoted by $P_{t}=F\left(R_{2\times 2}\left(P_{t-1}-T_{2\times 1}1_{1\times n}\right)\cup p_{t}\right)$, where F is a filter function to smooth the points. In this article, a simple medium filter is selected. $R_{2\times 2}$, $T_{2\times 1}$ are the rotation and translation matrix of the vehicle mentioned in Section 5.2. Through traversing $P_{t}$, if distance jump happens on the main direction or its normal direction, which is fit from set p using Random Sample Consensus (RANSAC), the intersection part of the vacancy and the visual parking area is used to judge whether the parking area is vacancy or not, which is demonstrated in Figure 10.

## 7. Experiment

The experiments setup includes two parts: sensor placement and datasets. The databases used in our experiments were acquired by a 360 surrounding camera system and ultra-sonic sensors. The camera system contained four fisheye cameras with a resolution of 640 × 480@30 FPS. The panorama image resolution is 500 × 500. The eight front and rear ultra-sonic sensors cover a range of 20–350 cm with an FOV of 90 degrees. The four side ultra-sonic sensors cover a range of 20–500 cm with an FOV of 50 degrees. The vehicle speed during the experiments was around 0–20 km/h. The sensor placement was demonstrated in Figure 2. Since all the data has been collected from a real-time system with the real processors, sensor positions and acquisition frequency, it is confirmed that the experiment setup is equivalent to the real-time application. We choose three typical parking scenes to test the algorithm proposed in this paper, including underground parking lots with white and yellow lines (UG), ground daytime (GD) and ground nighttime (GN). UG has 537 parking areas with 227 vacancies. GD has 336 parking areas with 144 vacancies. GN has 124 parking areas with 98 vacancies. In order to test the robustness of our proposed algorithms. The datasets we use contain different light conditions, ground materials, parking mark colors and garage types. The proposed detection and tracking methods were implemented in C++ language, and their execution times were measured on both 3.19 GHz Intel Core i7-4700MQ CPU and a NVIDIA TX1. The parameters we used in the experiments are shown in Table 1.

**Figure 11:**
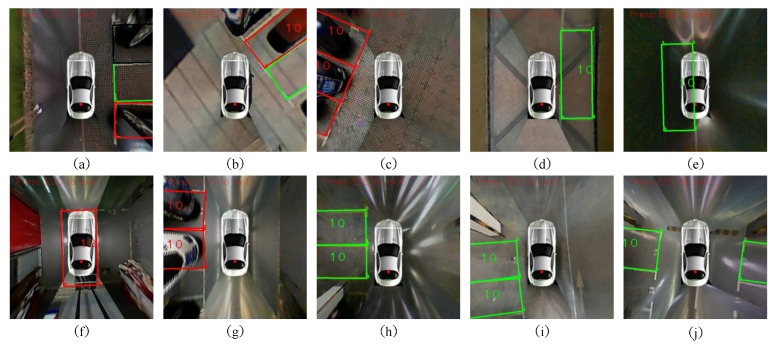
Parking area detecting results. The green box means the parking area is vacant and the red one means unparkable. (**a**–**e**) are the ground detecting results. The sceneries includes different ground materials and light conditions. (**e**) is the nighttime detecting results. (**f**–**j**) are the underground detecting results containing both white and yellow parking lines. The ground reflection and poor light condition are the biggest challenges for detection.

**Figure 12:**
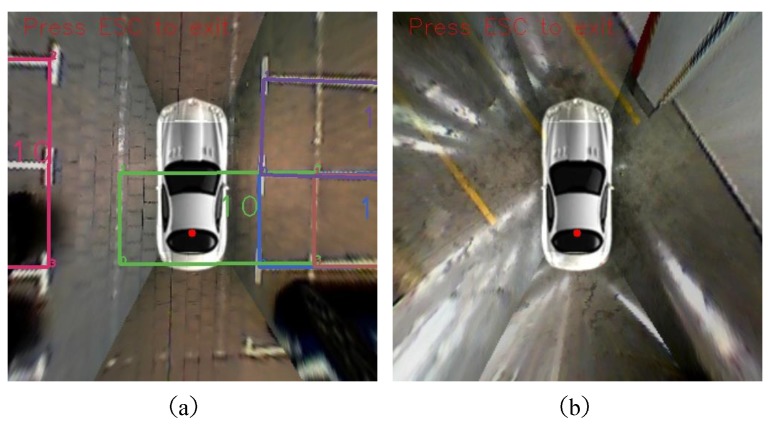
Typical failure modes. In (**a**), the left rose red one is caused by miss detection of one L-shaped structure which results in the detecting result as a parallel parking space. The right two error results are caused by disturbance lines incorrectly appearing on the ground. (**b**) is a situation that our proposed method currently does not support.

The detecting results of three datasets compared with ultra-sonic methods and pillar-based method in [13] are shown in Table 2, Table 3 and Table 4 and Figure 11. All the best results are highlighted in bold style. GD performs best among all of the datasets. The recall and precision are up to 0.9097 and 0.9632. Due to the ground reflection and some unclear marks, the detection result of UG are a little poorer than GD. Since our proposed algorithm is a vision-based method, poor light conditions in GN caused much more miss-detection than the other two datasets. Thanks to the headlight and reduction of speed, our proposed fusion algorithms are proven to work much better than conventional ultra-sonic methods. The recall and precision are 0.7959 and 0.9398, respectively. We have realized the pillar-based methods proposed in [13] to evaluate on our three datasets. Since this method is only adaptive to indoor and underground environments, GD gets the highest performance. The recall evaluated on our datasets are 0.8018, 0.7719 and 0.4388. There are three primary reasons causing this result. First, in our datasets, the pillow structure is different from that in [13]. Guild lines in [13] are solid lines, while we have dashed lines in most of the cases, which will cause failure in the “Guild Line Detection” part. Second, the ground reflection is much more serious in GD. The reflection causes many noise points in gradient calculation. Although RANSAC is utilized, the miss detection is still inevitable. Consequently, treating every L-shaped structure as the separate subassembly of one parking area in our proposed method shows much better robustness when some L-shaped structure fails to be detected. Usually, L-shaped structure of the outdo/or parallel parking areas are even farther from each other with disturbance of shadow. In addition, the assumption of opposite gradient direction does not work in the situations demonstrated in Figure 11c. However, since our proposed method needs to be adaptive and robust to different environments, our precision on three datasets are 0.9381, 0.9632, 0.9398, respectively and method in [13] are 0.9783, 0.9703, 0.9677, respectively. This is one of the limitations of our method. Figure 12 also shows the circumstances that our method currently does not support. There are three errors in (a) including the left one caused by miss detection of the L-shaped structure and the right two false positives caused by disturbance white lines on the ground. Fortunately, the left false positive is eliminated by ultra-sonic sensor fusion method.

**Figure 13:**
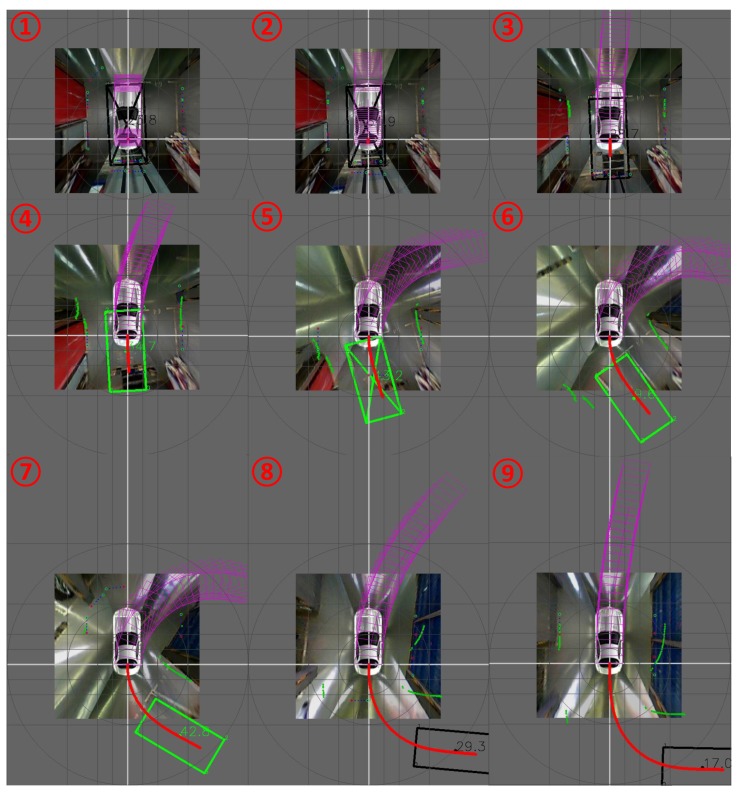
Parking area tracking results. The black rectangles represent vacant. The black rectangles represent unknown parkable status. In the first two frames, the unknown status is because the ego vehicle is inside the parking area and the ultra-sonic sensors could not scan this place. In the last two frames, the unknown status is caused by the distance out of scan range. The cross in a rectangle means a new detecting happened this moment. The number in the middle of a rectangle is the confidence score.

**Figure 14:**
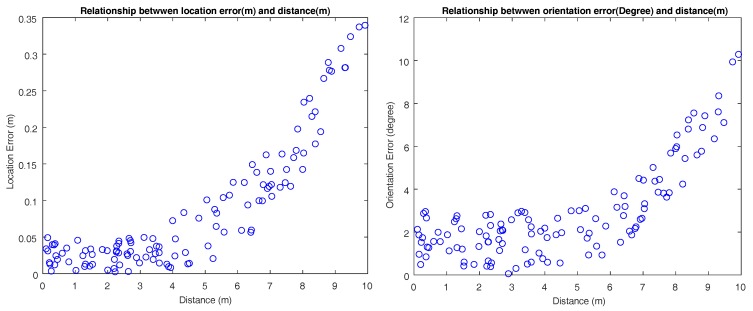
**Left**: location error of 120 samples. **Right**: orientation error of 120 samples.

Figure 13 shows a typical tracking period. With the help of a vehicle model, the parking area can be tracked even if all of the key L-shaped structures are lost. A rectangle without a cross in it means that it is a pure tracking result. The number in the middle indicates the confidence score of every frame. The score is continuously reducing when tracking in case a new detection has occurred. Since it is difficult to measure error when the vehicle is moving, we stop the vehicle to do the measurement. The relationship between error and distance from the ego vehicle is shown in Figure 14. The average location and orientation error are 8.5 cm and $2.8^{{}^{\circ}}$, and the max location and orientation error are 33.9 cm and $10.2^{{}^{\circ}}$. Error mostly comes from the image mosaicking and vehicle motion tracking model. The max error always happens when the parking area is far away from the vehicle, and the error usually increases with an increase in the distance between the ego vehicle and the parking area. In practical use, the accuracy of detecting and tracking is important, especially when the ego vehicle is close to the parking space. So, we just evaluate the distance from about 0–10 m.

The processing time of one frame is about 50 ms on Intel CPU and 135 ms on NVIDIA TX1 on average. About half of the processing time is caused by image mosaicking. A longer delay may cause a bigger error, so speed compensation using the processing time is a must in practical use. The value of vehicle speed multiplied by the time of processing is added to the longitudinal coordinates of four paring area points to compensate the quantity of position error.

## 8. Conclusions and Future Work

In this paper, we proposed a robust parking area detecting and tracking method by fusion of 360 surrounding cameras, an ultra-sonic method and a vehicle model. The novelty of the approach relies in its great adaptability to different environments and low computational cost. The algorithms are adaptive to most regular indoor and outdoor situations, and obtain a satisfying result. The 360 surrounding images can be easily stitched together through automatic calibration in the practical deployment. An L-shaped marks-based searching method is robust to most of the situations in China. The experiments show that both of the recall and precision are high and close to each other on different datasets. The similar performance also proves our method to be robust. A vehicle model-based tracking method not only balances the accuracy and computation complexity, but also accommodates both indoor and outdoor situations. Fusion-based vacancy detection is approved by one of the biggest car manufacturers in China and proves to be an accurate and low cost method for volume production.

Since parking lots and parking marks differ a lot from each other worldwide, it is necessary to build an adequate public dataset including different parking marks, ground materials, light conditions, etc. with marked ground truth vacant and occupied parking marks. Our team is now working through this and hopes to open access to the public soon. With the public dataset, comparison between different algorithms can be more meaningful. In addition, with large datasets, deep learning method and 3D information are what we will add to the system to make the improvement.

## Figures and Tables

**Table 1:** Parameters chosen for the experiments.

Minimum width of vertical parking area	2.2 m	Maximum width of vertical parking area	3.5 m
Minimum length of vertical parking area	5.1 m	Maximum length of vertical parking area	6.5 m
Minimum width of parallel parking area	2.1 m	Maximum width of parallel parking area	2.7 m
Minimum length of parallel parking area	5.3 m	Maximum length of parallel parking area	7.0 m
Scale of LSD API in OpenCV [30]	0.5	Sigma_scale of LSD API in OpenCV	0.375
Shape anlge of vertical parking area	$54^{{}^{\circ}}$ $90^{{}^{\circ}}$	Number of line angle group	10
Minimum width of parking edge	4 px	Minimum width of parking edge	13 px
Maximum line distance for combination of two LSD result	3 px	Angle tolerance of L-shaped extractor	$10^{{}^{\circ}}$
Minimum length of a valid LSD line after combination	15 px	Maximum length of a valid LSD line after combination	250 px
Color threshold ϵ in Section 5.1	5	Color threshold ξ in Section 5.1	150
Maximum distance for treating two line as intersection	10 px	Maximum point distance for treating two parking areas as the same	0.7 m

**Table 2:** Performance comparison of parking area detection methods in UG.

Method	No. of Vacant Parking Areas	No. of Correct Detection	No. of False Detection	Recall	Precision
Ultrasonic sensor-based method	227	90	19	0.3965	0.8257
Pillar-based method in [13]	227	182	**4**	0.8018	**0.9785**
Proposed fusion method	227	**197**	13	**0.8678**	0.9381

**Table 3:** Performance comparison of parking area detection methods in GD.

Method	No. of Vacant Parking Areas	No. of Correct Detection	No. of False Detection	Recall	Precision
Ultrasonic sensor-based method	144	62	7	0.4306	0.8986
Pillar-based method in [13]	114	88	**3**	0.7719	**0.9670**
Proposed fusion method	144	**131**	5	**0.9097**	0.9632

**Table 4:** Performance comparison of parking area detection methods in GN.

Method	No. of Vacant Parking Areas	No. of Correct Detection	No. of False Detection	Recall	Precision
Ultrasonic sensor-based method	98	41	1	0.4184	0.8238
Pillar-based method in [13]	98	43	**1**	0.4388	**0.9773**
Proposed fusion method	98	**78**	5	**0.7959**	0.9398
